# Is obesity the missing link? Ultra-processed foods and metabolic and reproductive outcomes: a narrative review

**DOI:** 10.3389/fnut.2026.1800945

**Published:** 2026-04-29

**Authors:** Noha M. Almoraie, Wajd D. Alomari

**Affiliations:** Department of Food and Nutrition, Faculty of Human Sciences and Design, King Abdulaziz University, Jeddah, Saudi Arabia

**Keywords:** diet quality, inflammation, metabolic, obesity, ultra-processed food

## Abstract

Ultra-processed food (UPF) has been extensively linked to obesity, diabetes, hypertension, metabolic disease, and cancer. It also contributes to the development of inflammation, oxidative stress, and other disease pathways, with obesity being a major concern. In this review, we offer insights into the role of UPF consumption on nutritional behavior, diet quality, and health. We also raise the questions of whether UPF promotes obesity that subsequently mediates the development of infertility, metabolic syndrome, nonalcoholic fatty liver disease (NAFLD), and hypertension, and whether UPF independently drives these conditions through obesity-independent pathways. Understanding the impact of UPF on diet quality and health can guide strategies to reduce its consumption and address related public health and sustainability challenges.

## 1 Introduction

Food processing and its relationship with health is a significant concern owing to its impact on the nutritional food profile. In response to the growing need to categorize foods based on their degree of processing, several classification systems have been developed. Among the most widely applied in nutrition research are those proposed by NOVA, the International Agency for Research on Cancer (IARC), the International Food Information Council (IFIC), and the University of North Carolina (UNC) (1). While some systems classify foods according to the extent and type of processing, others emphasize differences in formulation and ingredient composition. The NOVA food classification system defines ultra-processed food (UPF) as “industrial formulations of components derived from foods that contain hydrogenated oils and cosmetic additives (such as flavors and colors).” UPF may also contain maltodextrin, protein isolates, modified starch, and little, if any, raw foods Moreover, aggressive marketing methods, such as price reductions for super-sized services increase the consumption of these products (2). Recently, nutritional transition has resulted in a global shift away from consuming minimally processed foods and toward ultra-processed alternatives. The incidence of excess body weight and obesity is increasing owing to changes in the global food system, particularly in UPF (3, 4). Therefore, a dissonance exists, reflecting a change in the food and nutritional model, which reveals a series of epidemiological problems, including the rising incidence of diseases of civilization, such as obesity, diabetes, and heart disease (5). Furthermore, various studies have reported the profound influence of UPF patterns on the nutritional habits (3). These patterns are associated with higher intake of highly processed meats, refined grains, and fast foods (1). Urgent action is required to develop new public health strategies to prevent the progressive loss of traditional diets and raise awareness of the negative health effects of excessive UPF consumption. In this review, we aim to provide a brief overview of the current literature and key discussion points regarding UPF intake in the context of obesity-related diseases, focusing on inflammation, antioxidant status, obesity, diet quality, and lifestyle factors. It offers important insights into the relative roles of nutrient content compared with ultra-processing in obesity risk and how these factors interact with infertility, metabolic syndrome, non alcoholic fatty liver disease (NAFLD), and hypertension.

## 2 Food processing and the emergence of UPF

Food processing involves the transformation of basic components into edible or marketable meals through various physical, chemical, and mechanical processes (6). The food industry plays a vital role in providing safe, convenient, and shelf-stable food items to consumers globally (7). Food processing is instrumental in modern society, influencing dietary consumption as well as economic conditions, public health, and environmental sustainability. Throughout history, food processing and technology have greatly impacted the availability and composition of food. However, there is a growing need to simultaneously consider their health potential and environmental footprint (8). Food processing involves various methods used to convert raw or harvested food items into new products, ensuring their safety, palatability, and longevity (2, 9). Recently, notable shifts have been observed in food-processing methods to address evolving consumer preferences (10). The increased demand for foods with extended shelf lives and enhanced taste has resulted in the addition of various natural and artificial ingredients to processed foods (11, 12). This alteration in processing techniques may have implications for the nutritional value of these foods, which are often characterized by elevated levels of sugar, fat, and salt. The evolution of food production systems toward greater industrialization and the emergence of UPFs since the 1980s have brought about substantial changes in the human diet (2, 13). Previously dominated by traditional home-cooked meals made from minimally processed ingredients, modern diets now feature a considerable reliance on meals prepared outside the home using processed foods (14). From a sustainability perspective, future challenges in producing palatable, safe, healthy, and environmentally sustainable foods must be addressed (6). UPFs are often viewed unfavorably by segments of the scientific community, the public, and policymakers, and they are sometimes regarded as ‘not real food.’ In this context, understanding which specific technical procedures involved in food processing contribute to the reported negative consequences is essential. A more precise approach involves evaluating whether various processing methods are associated with food characteristics such as high glycemic index, accelerated eating rate, high energy density, or other factors, which can easily be correlated with negative health consequences (15). Fifteen original research studies and one review reported the effects of food processing methods on the chemical composition and functional properties of various food components. These methods include heat treatment, acid treatment, high-pressure homogenization, fermentation, frying, emulsification, sun-drying, extraction, and various separation techniques (7). As most consumers do not know much about these techniques, they may feel uncertain or concerned about the extent of food processing (16). Previously, food processing and nutrition sciences operated as distinct scientific domains. However, increasing concerns among policymakers, researchers, health professionals, consumers, and other stakeholders regarding the impact of food processing on health and well-being have prompted calls for integrated perspectives and pragmatic categorizations (13, 14). The fundamental driving force behind the development of food classification systems based on food processing is the growing concern regarding the impact of industrial food processing on diet quality, health, and the risk of developing chronic diseases (17). Much of the current literature focuses on UPF classification, and at least eight classification systems have been developed to categorize foods into distinct classes based on variables such as nature, degree, location, or goal of processing (14). Food processing and technology have historically shaped the availability and content of food, using methods that ensure safety, taste, and longevity. Recent shifts in processing, driven by consumer demand for longer shelf life and enhanced taste, often involve the addition of ingredients such as sugar, fat, and salt (18). This evolution has led to the rise of UPFs, which alter dietary patterns toward a reliance on processed foods prepared outside the home (19, 20). Concerns regarding the health impacts of food processing have prompted the development of food classification systems that aim to understand and address these issues (1).

## 3 UPF and diet quality

Although individual nutrient intake and food groups remain important components of healthy dietary patterns, other dimensions of dietary patterns are also crucial (21). The recent nutrition transition has resulted in a global shift away from consuming minimally processed foods toward ultra-processed alternatives (22) and from home-prepared dishes toward ready-to-eat meals and snacks (23). This period has also seen a rapid rise in the global prevalence of obesity in children and adults (24). Healthy dietary patterns, in addition to their nutrient content, such as the Mediterranean diet (MD), tend to be described as minimally processed (25). In contrast, unhealthy dietary patterns, such as the Western diet, tend to be ultra-processed (26). UPF remains a subject of ongoing debate, whether ultra-processed diets are detrimental to health owing to their poor nutritional quality, and whether the nature and extent of processing have health consequences remains inadequately explored (3).

Another critical feature of UPFs that results in excessive energy intake is their high energy density. In the United Kingdom, a national survey of 9,374 participants conducted over 6 years reported that the average consumption of UPF is ~34.9–80% of total caloric intake. Moreover, the highest percentage of dietary energy from UPF came from industrially packaged bread, packaged pre-prepared meals, sausages and other reconstituted meat products, confectionery, biscuits, pastries, buns, cakes, industrial chips (French fries), and soft and fruit drinks. This outcome is not accidental. Food technologists have employed advanced techniques in the design and manufacture of UPFs to make them appealing and enjoyable to eat (27). Overall, existing research points to UPFs as hyperpalatable and having a higher energy density than minimally processed foods. This reflects a broader nutritional transition from fresh, traditionally prepared meals to increased consumption of ready-to-eat, hyperpalatable food and beverage products.

UPF tends to be high in saturated fat, added sugar, and sodium (1), with meta-analyses demonstrating that high-UPF diets contain greater intakes of total energy, free sugars, and total and saturated fat, and lower intakes of fiber, protein, and some micronutrients (28, 29). A quantitative meta-analysis of nationally representative surveys on the consumption of UPF and the dietary and nutrient profiles of participants’ diets reported that among macronutrients, UPF consumption was linearly and positively associated with total and saturated fats and negatively associated with protein, fiber, and certain micronutrients, including potassium, magnesium, vitamin C (marginally), vitamin D, zinc, phosphorus, vitamin B12, and niacin (29). However, when considering the potential impact on health, diets lacking whole grains/dietary fiber and high in sodium are considered the risk factors most responsible for premature mortality (28). Numerous studies have highlighted the substantial influence of Western dietary preferences on the evolving nutritional behaviors of diverse demographics and shown a correlation with higher consumption of UPF, refined grains, fast food, and highly processed meat (30).

However, 14 nationally representative studies reported increased consumption of UPF along with their associations with diet quality and nutrient profiles. UPFs contribute ~21.5% of the total dietary intake in Brazil (31), around 47.7% in Canada (32), and about 30.2% of the daily energy intake in the United States (33). Similarly, UPFs account for a large proportion of dietary energy in Australia (42.0%) (34), Portugal (~24% among adults and 16% among older adults) (35), France (31.1% of total energy intake) (36), and Japan, where they contribute to at least one-third of the total energy intake among adults (37).

After adjusting for confounders, all 14 studies found that carbohydrate, added sugar, sodium content, and saturated fat content increased with an increase in UPF intake, alongside a linear inverse relationship with the dietary content of protein and fiber (27, 30–42). UPF consumption was linked to lower levels of essential nutrients, including vitamins B12, D, and E, as well as niacin, pyridoxine (vitamin B6), thiamine, riboflavin, copper, iron, phosphorus, magnesium, selenium, and zinc (30–42).

Although sodium and potassium intakes declined with higher UPF consumption, the sodium-to-potassium ratio remained elevated across all intake levels and increased progressively with greater UPF-derived energy contribution (36, 39, 40). Results showed strong associations between UPF consumption, unbalanced dietary nutrient profiles, and poor diet quality, which are predictive of increased risk of several diet-related diseases.

## 4 Obesity and related diseases

The rise of the global obesity epidemic has been widely recognized as being related to the increasing incidence of serious health risk factors and conditions (43), including insulin resistance, type 2 diabetes (44), NAFLD (45), and hypertension (46). Considering the serious health consequences linked to obesity, curbing the obesity epidemic is crucial for controlling the development of other metabolic conditions (43).

The major risk factors underlying obesity and its related disorders are consuming a high-calorie diet (47) and substitution of leisurely physical activities with sedentary activities, which ultimately results in excess energy stored in the body (48). Excessive lipid consumption from high-calorie diets, such as those rich in UPF, results in lipid accumulation in subcutaneous and visceral adipose tissues. Consequently, adipose tissue will be unable to store excess energy as triglycerides, leading the excess lipids to enter systemic circulation (3). Moreover, adopting a hypercaloric diet has been widely used to induce obesity in lab animals. Owing to its close resemblance to human obesity, such as excessive intake of high-calorie foods and a sedentary lifestyle, this model is highly valuable for studying obesity in laboratory animals (49). The high-fat diet (HFD) is known to be directly related to the development of obesity. In animals, chronic HFD feeding remains the most acceptable method for inducing obesity, liver damage, and NAFLD (50). Long-chain saturated fatty acids, primarily found in processed meat, are the most harmful lipids regarding the accumulation of adipose mass (51). As metabolic dysfunction and chronic inflammation intensify, obesity increases systemic oxidative stress and hormonal alterations, creating conditions that elevate the risk of cancer and fertility impairments (52). Evidence from previous studies shows that acute and chronic inflammation in people with obesity can lead to different complications such as hypertension, asthma, and type 2 diabetes (53, 54). Furthermore, inflammation is recognized as an underlying pathological and physiological process associated with increased levels of pro-inflammatory cytokines and white blood cells in the body (54). This condition is also central to vital processes such as tissue repair and wound healing. Akash et al. (55) found that inflammation occurs in the presence of pro-inflammatory cytokines and macrophages in body systems and tissues among patients with obesity. The authors further noted that these changes can be positive or negative, depending on the extent to which they affect the main body functions, such as blood circulation.

Among individuals with obesity and diabetes, insulin resistance is one of the most common complications, resulting from a complex relationship involving a wide range of molecular processes (44). The relationship between obesity and insulin resistance is complex and has been linked to a wide range of molecular processes. Evidence gathered from molecular studies has highlighted some of the fundamental mechanisms leading to the development of insulin resistance (56).

Results on insulin alterations are conflicting. Insulin has important metabolic effects in multiple organ systems. Insulin resistance is a physiological condition that occurs when insulin function and action become impaired. In such a state, organs such as the muscles and liver are sometimes incapable of responding to energy and metabolic demands. Research shows that complications can develop in several organs in the body and affect normal metabolic processes, such as oxidation and glucose uptake (57). In the long run, these changes affect the normal functioning of organs, such as the heart, liver, and pancreas (52, 58). Although the term “insulin resistance” is usually used to describe defective insulin-mediated glucose uptake in skeletal muscle, in the context of obesity and NAFLD, it additionally encompasses the liver, where insulin fails to adequately suppress glucose production, and adipose tissue, where lipolysis is insufficiently inhibited (59).

High-fat, high-caloric diets contribute to excessive weight gain and obesity, which in turn initiate a cascade of metabolic and endocrine disturbances that lead to several major chronic diseases (49, 50). As adiposity increases, altered adipokine secretion, low-grade inflammation, and insulin resistance disrupt metabolic homeostasis, creating the foundation for obesity-related hypertension through their effects on vascular function, fluid balance, and hormonal regulation (52). These same metabolic pathways also promote NAFLD by increasing hepatic fat accumulation and impairing lipid oxidation (45). Additionally, the endocrine disruption characteristic of obesity contributes to fertility impairments in men and women (60), illustrating how high-fat, high-calorie dietary patterns drive obesity and subsequently influence multiple organ systems and long-term health outcomes.

## 5 Discussion

### 5.1 Mechanisms linking UPF to weight gain and obesity

To better understand how certain foods contribute to obesity, examining the etiology of excessive food intake is essential. One essential feature of eating is that consumers have limited control over their food intake. Cohen and Farley argued that ‘.eating [is] an automatic behavior, as opposed to one that humans can self-regulate’. Many types of UPF bypass the appetite control system and induce excessive energy intake (61). A current proposal suggests that industrial food processing is the primary factor linking food to health outcomes (62). Furthermore, UPF contains higher levels of saturated fat, added sugar, and salt, but lower levels of fiber, minerals, and vitamins (28, 63). UPF may promote weight gain by displacing low-energy, nutrient-dense, unprocessed, and minimally processed foods and encouraging poor dietary habits (28, 64, 65). Their accessibility, low price, convenience, and aggressive marketing encourage involuntary overeating and continuous snacking, potentially replacing less processed and nutrient-dense foods in the diet (64, 66). High intensity flavoring in UPF may promote overeating and override endogenous satiety responses (67).

Previous studies have illustrated that diets rich in trans fatty acids and refined carbohydrates cause rapid and noticeable weight gain and obesity (68–71). UPF are frequently characterized by high levels of refined carbohydrates and fats, high energy density, and typically contain flavors and food additives that affect reward and satiety systems along the gut–brain axis; thus, a causal link with obesity is plausible” (72). Furthermore, high intake of UPFs alters lipid composition owing to the high presence of saturated and trans fatty acids, compromising the catabolism of very-low-density lipoproteins, increasing blood triglycerides, and reducing high-density lipoprotein (73). A randomized controlled trial investigating the effects of UPF on calorie intake and body weight revealed significant changes compared with unprocessed diets. In a study by Hall et al., participants consuming a UPF-based diet ate an average of 500 additional calories daily. Over 2 weeks, they gained 0.9 kg, whereas those on an unprocessed diet lost 0.9 kg. This highlights the importance of reducing UPF intake in obesity prevention strategies (74). A systematic review that included 10 cross-sectional and 13 prospective cohort studies reported that high consumption of UPF is linked to a higher risk of overweight and obesity and a poor cardiometabolic risk profile. The same review reported that individuals who consume more UPFs have an increased risk of developing cardiovascular disease, depression, and all-cause mortality (75). The characteristics of UPFs, combined with faster eating rates and high energy density, may contribute to their overconsumption and subsequent weight gain (76). Increased energy intake during the UPF period was attributable to significantly higher carbohydrate and fat consumption (74, 76).

### 5.2 Examining UPF consumption in relation to obesity-related biomarkers

Obesity is a considerable public health concern, contributing to serious health problems and diminished quality of life while placing a substantial burden on healthcare systems because of the increased demand for medical care and treatment of obesity-related conditions (77–79). Obesity is defined as excess body fat, indicated by a body mass index (BMI) > 30 kg/m^2^ or waist circumference (WC) > 88 cm in females or > 102 cm in males (80), although a call for alternative methodology to define obesity is rising (81). Growing awareness of the impact of UPF on health, along with its association as a risk factor for diet-related diseases, disorders, and conditions, is emerging rapidly. Recent technological advancements have substantially transformed the food production chain, increasing the accessibility and commercialization of UPF (76). These changes alter the nutritional content and sensory attributes of foods (2, 82). UPF are nutritionally ‘empty’ and often contain added substances such as sugars, salt, maltodextrins, protein isolates, artificial sweeteners, high-fructose syrups, and diverse additives, including colorants, flavorings, and thickeners (1). These ingredients are typically used to enhance the flavor of the products, making them more palatable, convenient, and economically accessible (4, 20). The link between food processing and the obesity epidemic has gained traction and has been validated in studies involving over 1 million participants (17). This led to the development of the NOVA food classification system, which categorizes foods based on their level of processing and identifies dietary factors associated with obesity risk (33, 83, 84).

Different cross-sectional studies using nationally representative samples have explored the association between UPF consumption and obesity. In Brazil, da Louzada et al. (85) found that household energy availability of UPF (purchased food items converted to kcal/day) was directly associated with average BMI and the prevalence of excess weight or obesity. As UPF consumption increased from Quintile 1 to 5, participants in the highest quintile had significantly higher BMI (0.94 kg/m2; 95% confidence interval [CI]: 0.42, 1.47), greater odds of being obese (odds ratio [OR] = 1.98; 95% CI: 1.26, 3.12), and excess weight (OR = 1.26; 95% CI: 0.95, 1.69) than those in the lowest quintile (85). In a second study in the United States, Juul et al., involving 15,977 participants, found that high intake of UPF was associated with a 1.61-unit higher BMI (95% CI 1.11, 2.10), 4.07 cm greater WC (95% CI 2.94, 5.19), and higher odds of overweight, obesity, and abdominal obesity (86). In Canada, Nardocci et al. (87) reported that individuals in the highest quintile of UPF intake by percent energy had a greater risk of obesity and overweight (BMI: 25–30) than those in the lowest quintile (87). In Korean adults aged 19–64 years from the Korea National Health and Nutrition Examination Survey 2016–2018, Sung et al. found that individuals in the highest quartile of UPF intake by percent energy had higher BMI and WC, with associations more pronounced in women (88). In a UK study of 6,143 adults aged 19–96 years, Rauber et al. found that individuals in the highest quartile of UPF intake by percent energy had higher BMI and WC and greater odds of having obesity than those in the lowest quartile (89).

### 5.3 Metabolic and endocrine impacts of UPFs in the context of obesity

UPF consumption is associated with obesity through mechanisms that extend beyond excess calorie consumption, influencing metabolic pathways and endocrine signaling (3, 4). Despite the growing evidence, the mechanistic basis through which UPFs contribute to metabolic disease risk remains unclear; however, UPFs can induce metabolic dysregulation through several interconnected processes (90).

The primary risk factors for developing metabolic disease are alcohol abuse, smoking, physical inactivity, and poor dietary habits (90). Shifts in population eating patterns have increased metabolic disease risk owing to the increased consumption of foods subjected to a high level of industrial processing (2, 7, 8), which consequently alters the foods’ natural composition to extend shelf life and enhance sensory properties, such as color, aroma, and flavor (1). Studies have shown that a higher degree of food processing is correlated with metabolic disease (3, 90). This association is attributed to the high content of saturated fat, trans-fatty acids, refined sugars, as well as additives, little or no fiber, vitamins, and minerals (28, 29). These substances can alter gut microbial communities, leading to an inflammatory environment that disrupts glucose metabolism. Thus, UPFs can influence metabolic physiology without excessive caloric intake by increasing low-grade inflammation, which interferes with mitochondrial function and reduces energy expenditure (90, 91). Additionally, these substances might contribute to the development of hepatic steatosis and other metabolic disease risks. UPFs are particularly endocrine-disrupting chemicals (EDCs) that interfere with hormonal regulation, immune responses, and microbial balance (92). Endocrine disruptors are believed to play a role in the pathogenesis of obesity and NAFLD (93). EDCs such as phthalates, bisphenols, and nitrates mostly originate from food processing, packaging, or storage. Studies continue to show that exposure to EDCs via UPFs may lead to hormonal perturbations in estrogenic, androgenic, and glucocorticoid pathways (92, 94). This disruption can result in metabolic diseases and hormone-related cancers due to the packaging of these foods, which may introduce contaminants such as bisphenols and phthalates, as well as additives and raw ingredients that could also be tainted. A high consumption of UPFs has been linked to elevated urinary levels of phthalate and bisphenol metabolites, implying higher exposure to these EDCs. Baker et al. (94) reported that a higher intake of UPF was associated with higher concentrations of di(2-ethylhexyl) phthalate metabolites among pregnant women, indicating that UPF intake may also play a role in socioeconomic inequalities in phthalate exposure. A study by Warkentin et al. (95), which included 640 women from the Barcelona Life Study Cohort, reported that plant-based diets can play a role in limiting exposure to some EDCs, but diets high in processed meat and UPFs may increase exposure during pregnancy. However, Martínez Steele et al. (96) conducted a study in a nationally typical group of the US population and found that urine levels of different phthalates and bisphenols increased with more intake of UPFs.

Importantly, obesity itself is characterized by chronic low-grade inflammation, altered adipokine secretion, and hormonal imbalances, which may further mediate the impact of UPFs on metabolic and reproductive health (97). Studies indicate that UPF-induced alterations in adiposity may partially explain these relationships (90, 98). Beyond excess adiposity, UPFs may exert indirect metabolic effects through gut microbiota dysbiosis, with evidence showing that higher UPF intake is associated with shifts in microbial taxa linked to inflammatory gastrointestinal conditions and low dietary fiber intake (99). UPF promotes obesity through more than just caloric excess. Harmful substances, such as Bisphenol A and phthalates, can leach from food containers and can linings, particularly into the high-fat and acidic foods common in UPFs (94). Certain dietary additives, including specific emulsifiers and preservatives, have also been shown to disrupt the gut barrier, facilitating the systemic absorption of both EDCs and inflammatory bacterial products (92, 94).

EDCs activate the PPARγ receptor, which triggers the differentiation of stem cells into adipocytes. This increase in fat mass then acts as a mediator for systemic inflammation. Adipose tissue expanded by EDCs secretes higher levels of pro-inflammatory cytokines, which interfere with insulin signaling, thereby mediating the development of type 2 diabetes (100, 101). Many EDCs are lipophilic, and they are stored in adipose tissue. Obesity, therefore, acts as a long-term reservoir for these toxins. This leads to a persistent, low-level release of EDCs into the bloodstream, which continues to disrupt endocrine signaling long after the initial exposure (102).

UPF facilitates obesity through both energy-dense foods and chemical disruption. Obesity then functions as a systemic mediator, both by amplifying chronic inflammation and by serving as a storage site for EDCs, which together drive the global rise in endocrine and metabolic diseases (100).

Moreover, individuals with metabolically unhealthy obesity exhibit higher circulating levels of EDCs compared to their metabolically healthy counterparts, and these levels may persist or even increase during weight loss, potentially contributing to weight regain and reductions in resting metabolic rate (103). These findings suggest that EDC exposure may not only promote obesity development but also sustain metabolic and endocrine dysregulation through adiposity-related mechanisms, including altered adipokine signaling and hormonal imbalance.

Understanding these pathways may clarify the mechanisms linking UPF to obesity and open opportunities for targeted interventions that address the underlying metabolic and endocrine disruptions.

### 5.4 Hypertension and UPF consumption in relation to obesity

Excessive salt intake is associated with high blood pressure in diverse human populations (104). Most sodium in modern diets comes from processed and packaged foods, not the saltshaker (8). Potential mechanisms have been hypothesized to explain the relationship between UPF and chronic diseases, including hypertension (8, 20). Studies with national representativeness conducted in several countries have identified changes in eating patterns (30–42). Among previous studies, an inverse association was observed between UPF consumption and potassium levels, as well as dietary fiber, alongside increased intake of free sugars, total fats, saturated fats, and trans fats, and an elevated sodium-to-potassium ratio (27, 30–42). Moreover, increased dietary sodium intake is associated with a higher risk of central and general obesity, and the urinary sodium-to-potassium ratio was also associated with an increased risk of hypertension, specifically in overweight individuals (105).

Recently, different studies reported that lower sodium intake combined with sufficient potassium lowers blood pressure (106–108). Several mechanisms have been identified by which sodium and potassium can influence blood pressure, and evidence indicates that the interaction between these nutrients plays a dominant role in the development of primary hypertension. In particular, the modern Western diet and UPF, which are high in sodium and low in potassium, produce a biological interaction with the kidneys, resulting in excessive sodium and insufficient potassium concentrations in the human body (28, 108). These biological changes result in vascular smooth muscle cell contraction, followed by an increase in peripheral vascular resistance and higher blood pressure, promote weight gain, insulin resistance, systemic inflammation, and endothelial dysfunction, all of which are key mechanisms involved in blood pressure elevation. However, the influence of sodium or potassium intake on the renin-angiotensin system, arterial stiffness, and endothelial dysfunction remains unclear (109).

However, a recent longitudinal analysis conducted used data from 5,957 adults in the REGARDS (Reasons for Geographic and Racial Disparities in Stroke) cohort who were free of hypertension at baseline (2003–2007) and followed up in 2013–2016 to examine the association between UPF consumption and incident hypertension. The study reported that during follow-up, 36% of the participants developed hypertension, and higher UPF consumption showed a positive linear association with hypertension incidence. In aggregate analyses, participants in the highest UPF quartile had 23% higher odds of developing hypertension than those in the lowest quartile (110). Similar results were found in national studies that revealed the association between UPF consumption and incident hypertension, greater increases in systolic and diastolic blood pressure over time, and a significantly increased risk of developing hypertension. Individuals in the highest UPF intake categories are more likely to develop high blood pressure compared to those in the lowest categories (1, 87, 111, 112). These findings were consistent across sensitivity and subgroup analyses, supporting the evidence that UPF-rich diets contribute to hypertension risk and recommending limiting UPF intake for cardiovascular health promotion (111–113).

Dietary patterns such as the Dietary Approaches to Stop Hypertension (DASH) and the Mediterranean diet play a critical role in the development and prevention of hypertension (114). Evidence consistently shows that healthy dietary patterns (an unprocessed diet) are strongly associated with lower blood pressure and improved cardiometabolic health. In contrast, a modern Western dietary pattern, such as high intakes of UPF, red and processed meats, refined carbohydrates, added sugars, and saturated fats, has been repeatedly linked to an increased risk of hypertension and other cardiometabolic disorders (2, 87, 111–113). Moreover, excessive consumption of UPF strongly contributes to obesity, a major independent risk factor for hypertension, thereby amplifying cardiovascular risk directly and indirectly (3, 4, 46, 51).

Recent studies have shed light on the notion that diets rich in UPF increase the likelihood of developing hypertension, whereas adherence to healthier dietary patterns, such as the DASH diet, significantly reduces blood pressure and the risk of hypertension (114, 115). Collectively, these findings underscore the pivotal role of dietary quality in blood pressure regulation and highlight UPF consumption as a modifiable risk factor linking poor diet quality, obesity, and hypertension.

### 5.5 UPF and NAFLD, oxidative stress, and inflammation

Obesity, including diabetes, hyperinsulinemia, insulin resistance, hyperlipidemia, and NAFLD, has been extensively linked to UPF (3, 93, 116). A meta-analysis included a total of 381,655 participants, and it found that obesity independently led to a 3.5-fold increased risk of developing NAFLD. Meta-analysis also suggested an obvious dose-dependent relationship between BMI and NAFLD risk (per 1-unit increment in BMI: RR = 1.20) (117). NAFLD can be best described as a metabolic dysfunction that stems from insulin resistance-induced hepatic lipogenesis. This lipogenesis increases oxidative stress and hepatic inflammation and is often potentiated by genetic and gut microbiome dysfunction (118, 119). Globally, NAFLD affects a substantial proportion of the population, with a higher prevalence observed in men (36.6%) than in women (25.5%). Although NAFLD prevalence was higher among individuals with obesity (57.5%) compared with non-obese adults (30.2%). A similar pattern is observed in children, where NAFLD prevalence is considerably higher among those with obesity (38.0%) than among their non-obese counterparts (14.3%) (120).

**Figure 1:**
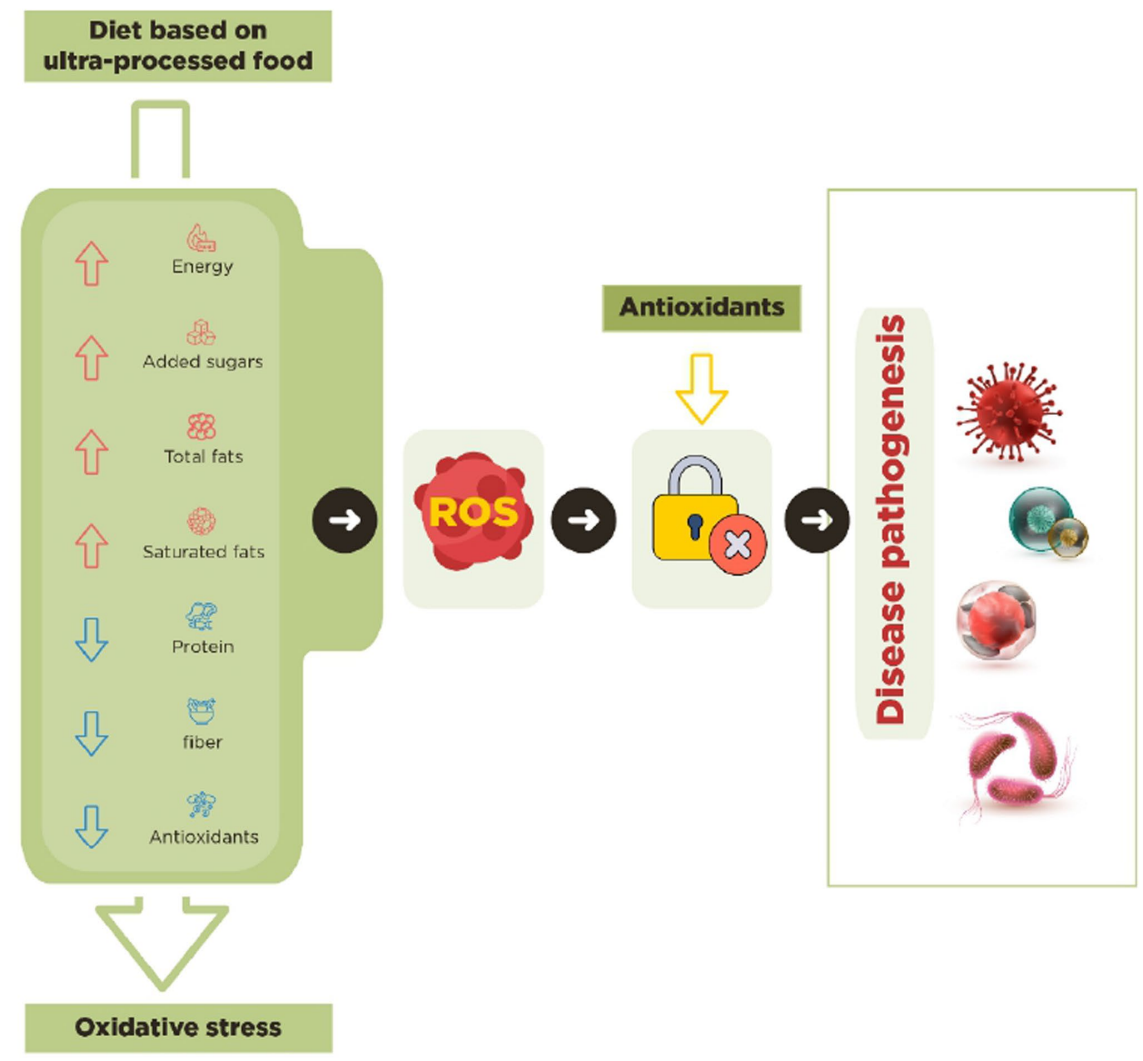
Ultra-processed foods contribute to oxidative stress by disturbing the balance between ROS production and the body’s antioxidant response.

Recent literature highlights that UPF may contribute to systemic inflammation owing to their high-fat content and effects on triggering oxidative stress (Figure 1) (121, 122).

Western dietary patterns, such as UPF, have been associated with an increased risk of chronic diseases and contribute to the development of these diseases through oxidative stress (75). An excess of reactive oxygen species (ROS) relative to antioxidant defenses leads to oxidative stress. This imbalance can cause damage to a broad spectrum of molecular species, including lipids, proteins, and nucleic acids (123). Furthermore, the diet contains milder antioxidants that also contribute to the body’s defense against oxidative stress. Although the body possesses numerous enzyme systems that neutralize free radicals, the most important dietary micronutrient antioxidants include vitamin E (tocopherol), vitamin C (ascorbic acid), and beta-carotene (124). Since the body cannot produce these micronutrients, they must be consumed. In contrast, high UPF intake, which could displace the intake of healthy antioxidant-rich foods, could be related to an increase in the pro-oxidative status (33, 40). The primary sources of dietary antioxidants are fruits, vegetables, whole grains, and nuts (122). However, UPF does not contain these foods and essential nutrients but contains high amounts of carbohydrates, free sugars, total fats, saturated fats, and sodium. This pattern also leads to lower fiber intake, indicating that individuals consuming UPF-rich diets are more likely to have low levels of antioxidants. Several studies have shown that individuals who consume UPF have lower levels of vitamin C, vitamin E, and fiber than those who consume unprocessed or minimally processed foods. Similar results were observed in other nationally representative studies, where increased UPF intake was associated with higher free sugar and lower intakes of vitamin C, beta-carotene, vitamin E, fiber, and protein (31–42).

UPF consumption is associated with lower antioxidant status in the body, as measured by the levels of antioxidant enzymes and biomarkers (124). This may contribute to the development of chronic diseases, as a low antioxidant status has been linked to increased oxidative stress and inflammation. Chronic inflammation can lead to the development of several long-term diseases, such as cardiovascular disease, type 2 diabetes, and cancer (125, 126). The composition of UPF―characterized by high daily energy intake, energy density, free sugars, and fats―is associated with persistent low-grade inflammation (3, 4, 127). A recent systematic review and dose–response meta-analysis involving 513,440 participants and 20,637 NAFLD cases reported that the highest UPF consumption was associated with a 22% increased risk of NAFLD, compared to the lowest consumption, as a 10% increment in UPF consumption was associated with a 6% higher risk of NAFLD. Dose–response analysis showed a linear trend association between UPF consumption and risk of NAFLD (93).

Several studies have shown that higher UPF intake is directly associated with dietary patterns high in calories, sweets, refined grains, red and processed meats, snacks, and sugary beverages. These patterns are linked to elevated levels of serum interleukin-6 (121), tumor necrosis factor-alpha (128), and C-reactive protein, a well-established marker of inflammation, suggesting that decreasing UPF intake can help reduce chronic inflammation (124).

### 5.6 Reproductive and fertility impairments associated with UPF in the context of obesity

Dietary patterns and exposure to potential EDCs play a fundamental role in influencing fertility by impacting various physiological and hormonal factors (129). Diets have shifted from traditional dietary patterns toward increased consumption of UPFs (1). Growing concern regarding infertility and human semen quality exists because globally, 8–12% of couples of reproductive ages are experiencing difficulties conceiving, with male-related factors accounting for nearly 40–50% of infertility cases (130). Recently, there has been a remarkable decrease in semen quality, particularly in developed and industrialized countries, which highlights the potential roles of environmental and lifestyle factors in this decline (130–132). The first line of evidence supporting the adverse impact of unhealthy diets on fertility involves proving the association between the Western diet and reproductive health and fertility. Unfortunately, the Western dietary pattern, which is associated with a higher consumption of UPF, has been rising during recent decades (133). The Western diet has been frequently associated with lower semen concentrations in males (134), lower progesterone levels, fewer available blastocysts, and lower pregnancy rates in females (135–138).

A recent review conducted to examine different dietary patterns and their impact on female and male fertility reported that processed foods are the primary dietary component of the Western diet most strongly associated with adverse reproductive outcomes (133). Two principal pathways have been proposed to explain the effect of UPF consumption on fertility. First, researchers assumed that the nature of the UPF affects reproductive and fertility. As previously approved, UPFs are industrial formulations. UPFs, known for their poor nutritional quality, are involved in mechanisms that hamper reproductive outcomes due to high levels of fats, especially TFAs, and added ingredients, including sugar, salt, fat, artificial colors, flavors, and stabilizers (31–40). Data on male and female fertility and UPF consumption represent a relatively new area of research (137–141). Recently, two studies conducted among females suggest that excessive intake of UPF is significantly associated with an increased risk of female infertility (140, 141). Plus, a diet with greater inflammatory potential, such as UPF, was associated with higher odds of self-reported fertility problems (140). Therefore, to improve reproductive health, women of childbearing age are advised to modify their diets and reduce UPF consumption (141).

Approximately 40–50% of cases recognize a male factor and may result from issues with sperm production, sperm function, or sperm delivery (142), which may explain why research on UPF consumption has more frequently focused on male reproductive outcomes (133, 138, 142). High UPF consumption was associated with a lower sperm concentration and progressive motility (143). A similar association was observed in another study, which reported that sperm concentration and sperm count were lower for each increase in UPF consumption (138).

Second, higher consumption of UPF may contribute to infertility indirectly through the development of obesity (144). The association between UPF and obesity has been well established, as diets rich in UPFs are strongly associated with excessive energy intake and weight gain, and obesity (85–89). Obesity is a well-established risk factor for impaired fertility due to its effects on hormonal regulation, insulin sensitivity, and inflammatory pathways (144). Therefore, studies assumed that obesity may act as a mediating factor linking high UPF consumption to reduced fertility outcomes (137, 145–147). Some studies have considered obesity (commonly assessed by BMI) as a potential mediator in the association between UPF consumption and infertility. Therefore, adjusted models are often constructed to account for its effect. For instance, one study reported no significant increase in the odds of female infertility among individuals with higher UPF consumption after adjusting for all covariates, including BMI. However, this relationship changed when BMI was excluded from the model. The results indicated that women in the highest tertile of UPF consumption had increased odds of infertility (147). This pattern suggests that BMI may mediate the relationship between UPF intake and infertility (137, 145, 146).

For instance, strong evidence exists that adherence to healthy dietary patterns rich in unprocessed or minimally processed foods, such as fruits, vegetables, legumes, or nuts, and low in red and processed meats or sugar-sweetened beverages confers significant health benefits. In line with this, a recent study was conducted to examine the correlation between adherence to the MD, as opposed to UPF intake, and the quality of sperm parameters in participants undergoing semen analysis. The study reported that adherence to MD, and conversely reduced intake of UPF, is associated with a better semen profile (148).

### 5.7 Challenges and future research directions

High UPF consumption is consistently linked to excess adiposity through mechanisms including high energy density, low satiety, rapid digestibility, and disruption of appetite regulation. These factors mediate increased risks of type 2 diabetes, metabolic syndrome, NAFLD, hypertension, and infertility. The association between UPF and obesity has been well established, although it remains unclear whether UPF promotes obesity that subsequently mediates the development of infertility, type 2 diabetes, metabolic syndrome, NAFLD, and hypertension, or whether UPF independently drives these conditions through obesity-independent pathways. Future research on UPF consumption and chronic diseases should consider individual variability, lifestyle factors as confounders, and the complex, multifactorial nature of obesity. Moreover, researchers examining the relationship between UPF intake and obesity-related health outcomes should carefully consider obesity as a potential confounding and/or mediating variable. To overcome challenges, future studies should be focused on long-term interventions to observe sustained impacts and engage in detailed mechanistic studies utilizing advanced techniques.

It is essential to acknowledge several limitations inherent to research on UPF consumption. First, although the NOVA classification system is widely used and forms the basis of this and many previous reviews to define UPFs, it has faced methodological criticism, particularly due to potential misclassification of foods and its limited capacity to fully reflect the complexity of food formulation and processing. Secondly, dietary assessment methods, especially food frequency questionnaires (FFQs), introduce further uncertainty due to recall bias, measurement error, and variability in how UPFs are categorized across studies. Moreover, heterogeneity in dietary assessment tools and definitions of UPF intake complicates comparisons between studies and may contribute to inconsistent findings. Finally, while the current body of evidence suggests meaningful associations between UPF consumption and adverse obesity-related diseases, caution is advised in inferring causality.

Further investigation should prioritize longitudinal and intervention-based studies to elucidate the causal mechanisms linking UPF consumption to adverse health outcomes.

## 6 Conclusion

In considering UPF over-consumption as an important risk factor for non-communicable diseases, overweight, and obesity, the results reinforce the importance of public-health strategies to improve the population’s health by promoting MD as a sustainable diet and limiting the intake of UPF. Evidence indicates that UPF adverse effects on metabolic, cardiometabolic, and reproductive health are only partially mediated by adiposity, with direct impacts through inflammation, insulin resistance, oxidative stress, and endocrine disruption. Elevated UPF intake correlates with increased consumption of unhealthy foods or poor dietary quality, such as refined grains, fast food, and highly processed meats. UPF is associated with being overweight or obese when combined with high levels of sedentary behavior. Mechanistic research is essential to clarify the causal pathways underlying the relationship between food processing and obesity. Trend diets seem to influence women’s and men’s fertility, as growing evidence shows that dietary patterns affect hormonal balance and reproductive function. UPF has been associated with increased inflammation, insulin resistance, and oxidative stress, all of which can impair ovulation, menstrual regularity, sperm concentration, and sperm motility. While adherence to balanced dietary patterns such as the MD or DASH diets has been associated with better reproductive outcomes, such as better semen parameters in men and fertility outcomes in women.
